# Evaluation of recombinant human erythropoietin responsiveness by measuring erythrocyte creatine content in haemodialysis patients

**DOI:** 10.1186/s12882-021-02623-4

**Published:** 2021-12-12

**Authors:** Shun Hasegawa, Seishi Nakamura, Tetsuro Sugiura, Yoshiaki Tsuka, Nobuyuki Takahashi, Koichiro Matsumura, Toshika Okumiya, Masato Baden, Ichiro Shiojima

**Affiliations:** 1 Department of Nephrology and Cardiology, Takarazuka Hospital, 2-1-2 Nogami, Takarazuka, Hyogo 665-0022 Japan; 2 The Second Department of Internal Medicine, Kansai Medical University, Osaka, Japan; 3 Department of Nephrology, Kansai Medical University Kori Hospital, Osaka, Japan; 4 Graduate School of Health Sciences, Kumamoto University, Kumamoto, Japan

**Keywords:** Erythrocyte creatine, Erythropoiesis, Erythropoiesis stimulating agent, Haemodialysis, Renal anaemia

## Abstract

**Background:**

One of the main causes of anaemia in patients with end-stage renal disease is relative deficiency in erythropoietin production. Eythropoiesis stimulating agent (ESA), a potent haematopoietic growth factor, is used to treat anaemia in haemodialysis patients. The effect of ESA is usually assessed by haematological indices such as red blood cell count, haemoglobin concentration and haematocrit, but erythrocyte indices do not provide information of the rapid change in erythropoietic activity. As erythrocyte creatine directly assess erythropoiesis, the aim of this study was to evaluate the effect of ESA in haemodialysis patients by measuring the erythrocyte creatine content.

**Methods:**

ESA dose was fixed 3 months prior to the enrollment and was maintained throughout the entire study period. Erythrocyte creatine was measured with haematologic indices in 83 haemodialysis patients. Haemoglobin was also measured 3 months after.

**Results:**

ESA dose (152.4 ± 62.9 vs. 82.2 ± 45.5 units/kg/week, *P* = 0.0001) and erythrocyte creatine (2.07 ± 0.73 vs. 1.60 ± 0.41 μmol/gHb, *p* = 0.0003) were significantly higher in 27 patients with haemoglobin <10 g/dL compared to 56 patients with haemoglobin ≥10 g/dL. There was a fair correlation between ESA dose and the concentration of creatine in the erythrocytes (r = 0.55, *P* < 0.0001). Increase in haemoglobin (>0.1 g/dL) was observed in 37 patients, whereas haemoglobin did not increase in 46 patients. Erythrocyte creatine levels were significantly higher in those patients with an increase in haemoglobin compared to those without (2.04 ± 0.64 vs. 1.52 ± 0.39 μmol/gHb, *p* < 0.0001). When 8 variables (ESA dose, erythropoietin resistance index, C-reactive protein, intact parathyroid hormone, iron supplementation, presence of anaemia, erythrocyte creatine and reticulocyte) were used in the multivariate logistic analysis, erythrocyte creatine levels emerged as the most important variable associated with increase in haemoglobin (Chi-square = 6.19, *P* = 0.01).

**Conclusion:**

Erythrocyte creatine, a useful marker of erythropoietic capacity, is a reliable marker to estimate ameliorative effectiveness of ESA in haemodialysis patients.

## Background

The primary cause of anaemia in patients with end-stage renal disease is inadequate erythropoiesis caused among others by insufficient production of erythropoietin [1]. Erythropoiesis stimulating agent (ESA), a potent haematopoietic growth factor, has been shown to be effective in correcting anaemia in chronic renal failure [2–5], but the response rate is variable because resistant factors of erythropoiesis such as iron deficiency, inflammation, aluminum intoxication, hyperparathyroidism, blood loss or bone marrow dysfunction are also involved [5, 6]. Early detection of poor ESA response is clinically important to increase the dose of ESA and eliminate transfusion requirements [7]. On the other hand, early detection of favorable response to a relatively low dose of ESA could save costs and avoid increased incidence of side effects. In contrast to haemoglobin levels which take a relatively long time to increase, total reticulocyte count can assess rapid change in erythropoiesis [8, 9]. However, total reticulocyte count has seldom been used in clinical practice, because it is generally considered to be inaccurate and imprecise [10, 11]. Since creatine content in the erythrocyte rapidly declines with erythrocyte aging, it is used to measure erythrocyte life span and erythropoietic capacity [12–14], but the clinical usefulness of erythrocyte creatine in patients receiving ESA has not yet been fully elucidated. Accordingly, we attempted to evaluate the ameliorative effectiveness of ESA in patients undergoing maintenance haemodialysis by measuring erythrocyte creatine content.

## Methods

### Study patients

We evaluated 83 patients (57 men and 26 women, mean age of 74 years) aged ≥20 years who had been receiving maintenance haemodialysis 3 times a week for at least 6 months in the Dialysis Unit of Takarazuka Hospital. All of our outpatients on haemodialysis were maintaining quality of life and took normal dialysis-diet with no extra creatine supplementation. The exclusion criteria were as follows: bleeding event within 3 months (n = 1), infection requiring parenteral antibiotics (n = 1) and mechanical heart valves (n = 2). None of the patients had concurrent malignancy, haemolytic disease or blood transfusion within 3 months.

### Study protocol

**Fig. 1:**
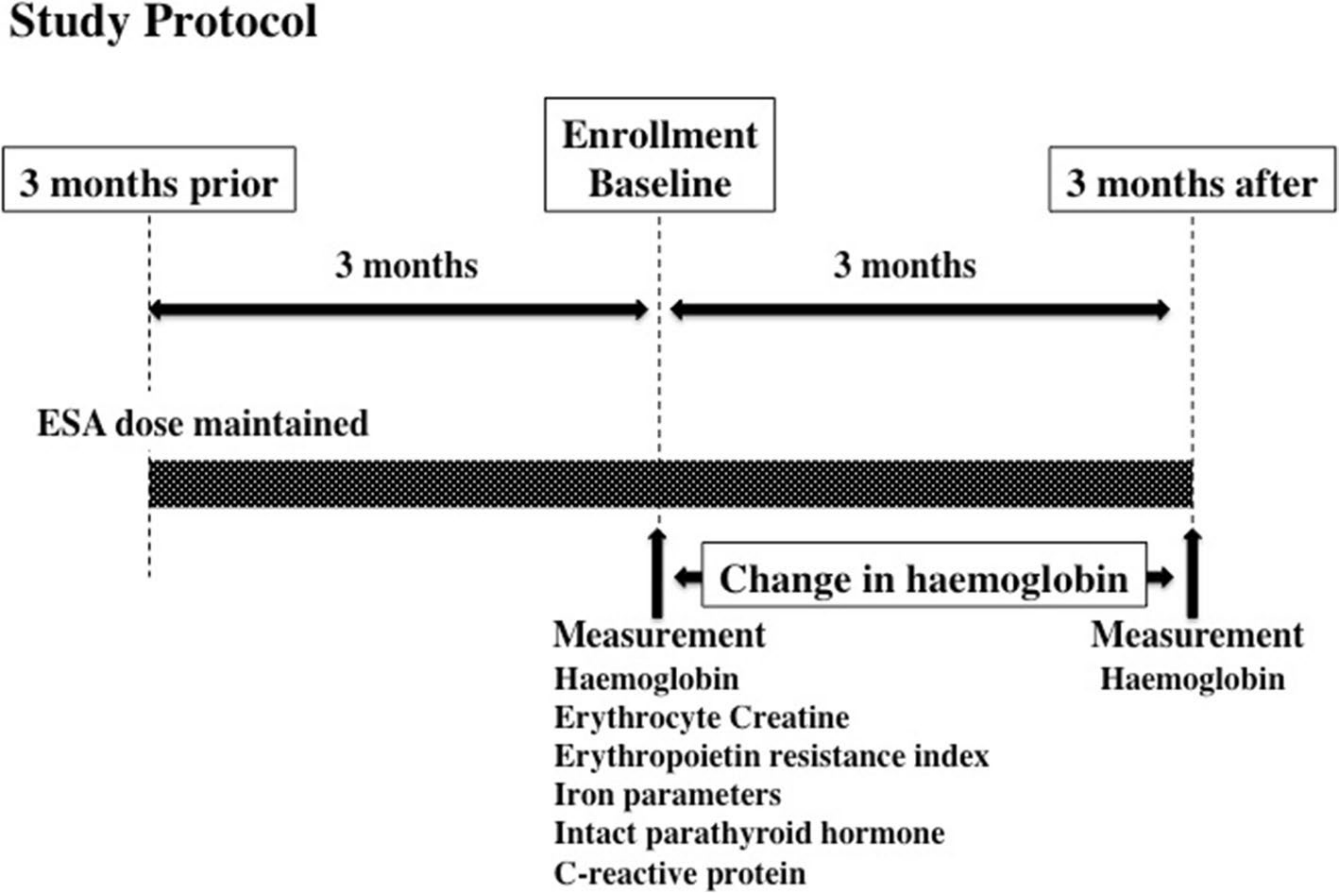
Study protocol. Erythropoiesis stimulating agent (ESA) treatment was fixed 3 months prior to the enrollment and was maintained during 6 months period. Laboratory tests were performed at baseline and haemoglobin was measured 3 months later

Study protocol is shown in Fig. 1. ESA treatment was fixed for a duration of 3 months prior to the enrollment and was maintained throughout the study period. Baseline blood examination including the erythrocyte creatine content and other laboratory examination (red blood cell count, haemoglobin, haematocrit, reticulocyte, haptoglobin, transferrin saturation, ferritin, intact parathyroid hormone, serum calcium, serum phosphorus, serum albumin and C-reactive protein) were performed. Intravenous iron treatment with 40 mg ferric saccharate 3 times a week was administered at the end of each haemodialysis session and was followed by 100 mg of oral iron supplements in patients with absolute iron deficiency, defined as transferrin saturation < 20% and serum ferritin <100 ng/ml [15, 16]. Patients were divided into 2 groups; patients with haemoglobin <10 g/dL and those with haemoglobin >10 g/dL according to the Guidelines of Japanese Society for Dialysis therapy [16] and clinical characteristics, haemodialysis conditions, treatments and laboratory indices were compared between the 2 groups. Haemoglobin was measured 3 months later and percent change in haemoglobin; (3 months minus baseline)/ baseline x 100% was calculated and expressed as percentage. The study protocol was approved by the Takarazuka Hospital ethical committee for human research. All the patients provided written informed consent, and the investigation conformed to the principles outlined in the Declaration of Helsinki.

### Haemodialysis

All patients were dialysed for 4.0–8.0 h, using a single-use dialyser: cellulose FB, Nipro Corporation, Osaka, Japan), poly-sulfone, (PN, Nikkiso Co., Ltd., Tokyo, Japan), polyethersulfone (PES, Nipro Corporation, Osaka, Japan), or polymethylmethacrylate (NF-H, Toray Medical Co., Ltd., Tokyo, Japan) with a 1.3–2.1 m^2^ effective surface area. All patients received haemodialysis with blood flow of 200 ml/min with dialysate flow of 500 ml/min. In all patients, haemodialysis was performed via native arteriovenous fistulas with a dual plastic needle and 16-gauge cannula. The patients in the haemodialysis group uniformly received a dialysate (D-dry, Nikkiso Co., Ltd., Tokyo, Japan) and an anticoagulant with heparin sodium. Bolus of heparin sodium 1000 units was intravenously administrated at the start of haemodialysis followed by continuous administration of 500 to 750 units/hour. The dialysate temperature of extracorporeal circulation was strictly maintained at 36–38 °C. ESA and iron therapy were prescribed in accordance with the Kidney Disease: Improving Global Outcomes Clinical Practice Guideline 2012 [15]. Erythropoietin therapy with epoetin beta pegol was administrated intravenously at the end of haemodialysis. Haemodialysis time (hours/week), intradialytic ultrafiltration rates (ml/hour/kg) were measured and Kt/V, as an index of urea clearance was calculated. These indices were calculated as the average of the values from 3 consecutive haemodialysis sessions. One of the following dialysis membranes was used: cellulose FB, Nipro Corporation, Osaka, Japan), poly-sulfone, (PN, Nikkiso Co., Ltd., Tokyo, Japan), polyethersulfone (PES, Nipro Corporation, Osaka, Japan), or polymethylmethacrylate (NF-H, Toray Medical Co., Ltd., Tokyo, Japan).

### Laboratory measurements

Blood samples were drawn immediately before the haemodialysis. Haematologic examinations and reticulocyte counts were carried out with a Sysmex XN 1000 (Sysmex, Kobe, Japan). Haptoglobin was measured by a TIA method with JCA-BM 6010 (JEOL, Tokyo, Japan). Serum iron was measured by a Nitroso-PSAP method with AU 5840 (Beckman Coulter; Tokyo, Japan), unsaturated iron binding capacity (UIBC) was by a Nitroso-PSAP method with BM-6050 (JEOL, Tokyo, Japan) and ferritin by radioimmunoassay with AU-5840 (Beckman Coulter, Tokyo, Japan) and transferrin saturation was calculated as [Serum iron/ (Serum iron + UIBC)] x100 (%). Intact parathyroid hormone was measured by the ECLIA method with Cobas 8000 (Roche Diagnostics, Tokyo, Japan). The other biochemical laboratory measurements were performed by TBA-120 FR automated biochemical analyzer (Canon, Osaka, Japan).

### Measurement of the erythrocyte creatine content

Erythrocyte creatine levels were assayed enzymatically in accordance with a previous report [17]. Measured date are expressed as micromole per gram of haemoglobin (μmol/g Hb). Briefly, blood samples were collected in ethylenediaminetetraacetic acid-containing tubes and centrifuged to remove the plasma and buffy coat. After lysis and deproteinisation of packed erythrocytes, the supernatant was obtained by centrifugation and filtration. The creatine concentration in the supernatant was measured using the enzymatic assay method. Erythrocyte creatine content represents the average or cumulative erythropoietic activity up to the present [18, 19].

### Statistical analyses

Results are expressed as mean ± standard deviation. Statistical analyses between the 2 groups were performed by one-way layout analysis of variance or chi-square analysis followed by Scheffe type multiple comparison method. Change in the haemoglobin from baseline to 3 months after was estimated by 2-way repeated measures ANOVA. Multivariate logistic regression analysis was performed to evaluate the important variables related to change in haemoglobin at 3 months. A probability value of <0.05 was considered significant. Parameters were compared with the use of commercially available statistical software (STATVIEW, Abacus Concepts, Berkeley, CA).

## Results

Clinical characteristics are shown in Table 1.

**Table 1:** Clinical characteristics

Number of patients	83
Age (years)	73.7 ± 14.5
Gender (male/female)	56/27
Diabetes mellitus (present/absent)	34/49
Height (cm)	162.0 ± 9.2
Weight (kg)	56.8 ± 14.0
Body surface area (m^**2**^**)**	1.60 ± 0.21
Haemodialysis time (hours/week)	16.0 ± 4.2
Intradialytic ultrafiltration rate (ml/hour/kg)	8.8 ± 3.0
Kt/v	1.72 ± 0.48

Data are represented as mean ± SD. *Kt/v* urea clearance

### Anaemia

**Fig. 2:**
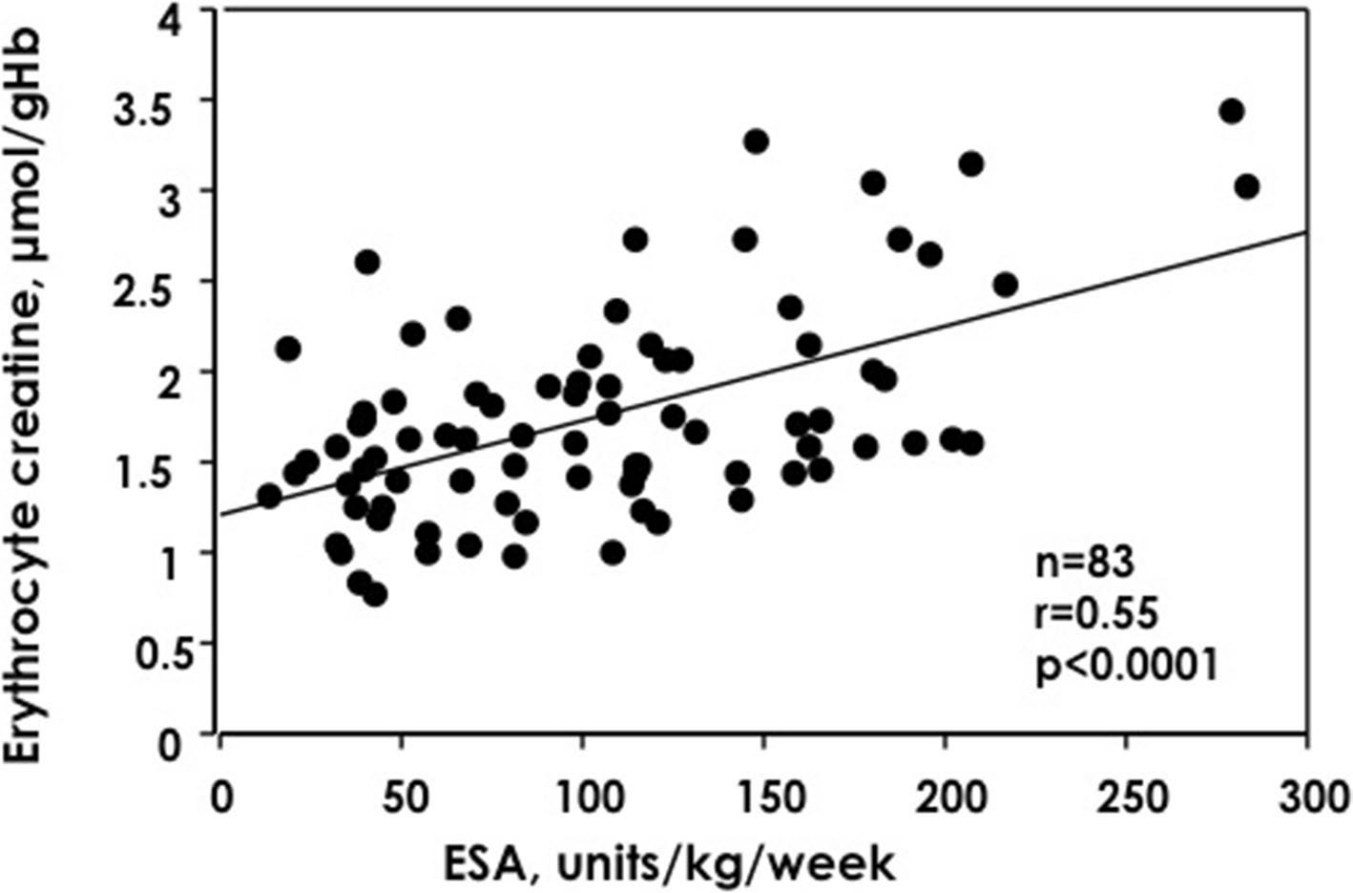
Relation between dose of erythropoiesis stimulating agent (ESA) and erythrocyte creatine

Twenty-seven patients (33%) had haemoglobin <10 g/dL and 56 patients had haemoglobin >10 g/dL. Patients with haemoglobin <10 g/dL received significantly higher ESA dose compared to those with haemoglobin <10 g/dL (Table 2). There were no significant differences in reticulocyte, haptoglobin, transferrin saturation, ferritin, incidence of iron deficiency, intact parathyroid hormone, serum calcium, serum phosphorus, albumin, C-reactive protein and frequency of iron supplementation between the 2 groups. However, erythrocyte creatine content, ESA dose and erythropoietin resistance index were significantly higher in patients with haemoglobin <10 g/dL compared to those with haemoglobin >10 g/dL. In this study, erythrocyte creatine had fair relations with erythropoietin resistant index (r = 0.59, *P* < 0.0001) and dose of ESA (r = 0.55, *P* < 0.0001) (Fig. 2) .

**Table 2:** Comparison between patients with and without anaemia

	Haemoglobin		*P* Value
	<10 g/dL (*n* = 27)	≥10 g/dL (*n* = 56)	
Age (years)	71.4 ± 13.5	71.9 ± 15.1	0.87
Gender (male/female)	14/13	42/14	0.05
Haemodialysis time (hours/week)	16.4 ± 4.1	15.7 ± 4.2	0.45
Intradialytic ultrafiltration rate (ml/hour/kg)	9.3 ± 3.1	8.6 ± 2.9	0.35
Kt/v	1.8 ± 0.5	1.7 ± 0.5	0.5
Erythrocyte creatine (μmol/gHb)	2.07 ± 0.73	1.60 ± 0.41	0.0003
**ESA dose** (units/kg/week)	152.4 ± 62.9	82.2 ± 45.5	0.0001
Erythropoietin resistance index (units/kg/week/g/dL)	17.6 ± 7.9	7.6 ± 4.4	0.0001
Red blood cell (x 10^4^μL)	288.0 ± 35.4	358.8 ± 33.1	0.0001
Haematocrit (%)	27.2 ± 4.4	34.5 ± 2.4	0.0001
Haemoglobin (g/dL)	8.9 ± 0.9	11.0 ± 0.7	0.0001
MCV	98.6 ± 6.7	97.2 ± 5.6	0.32
MCH	31.2 ± 2.4	30.9 ± 2.0	0.54
MCHC	31.6 ± 0.9	31.8 ± 0.7	0.42
Reticulocyte (%)	16.2 ± 5.1	15.1 ± 4.8	0.31
Haptoglobin (g/dL)	88.5 ± 51.1	97.3 ± 50.4	0.46
Transferrin saturation (%)	24.4 ± 12.9	29.5 ± 17.5	0.19
Ferritin (ng/mL)	110.4 ± 88.0	126.6 ± 114.8	0.52
Iron deficiency (present/ absent)	18/9 (67%)	29/27 (52%)	0.2
Iron supplement (present/ absent)	9/18 (33%)	13/43 (23%)	0.43
Intact parathyroid hormone (pg/mL)	146.7 ± 99.0	155.1 ± 104.2	0.73
Serum calcium (mg/dL)	9.5 ± 0.5	9.5 ± 0.5	0.98
Serum phosphorus (mg/dL)	4.5 ± 1.2	5.1 ± 1.4	0.085
Albumin (g/dL)	3.6 ± 0.4	3.6 ± 0.3	0.39
C-reactive protein (mg/dL)	0.35 ± 0.29	0.31 ± 0.37	0.61

Data are represented as mean ± SD. *ESA* Eythropoiesis stimulating agent, *Kt/v* Urea clearance, *MCH* Mean cell haemoglobin, *MCHC* Mean cell haemoglobin concentration, *MCV* Mean cell volume

### Change in haemoglobin

**Fig. 3:**
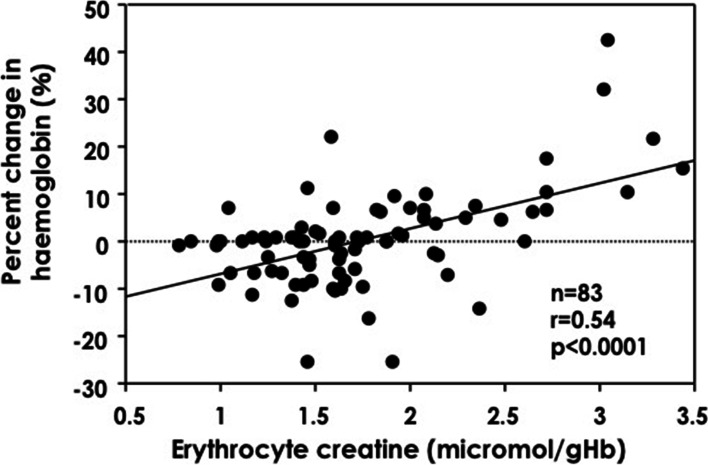
Relation between erythrocyte creatine and percent change in haemoglobin. Percent change in haemoglobin: 3 months minus baseline/ baseline x 100 (%)

Thirty-seven (45%) patients showed increase in haemoglobin (>0.1 g/dL) at 3 months, whereas haemoglobin did not increase in 46 patients. There were no significant differences in haptoglobin, transferrin saturation, ferritin, incidence of iron deficiency and intact parathyroid hormone between the 2 groups, but presence of anaemia, reticulocyte, erythropoietin resistance index, ESA dose, frequency of iron supplementation and erythrocyte creatine levels were significantly higher in patients with increased haemoglobin concentrations compared to those without (Table 3). Erythrocyte creatine had a fair correlation with percent change in haemoglobin (r = 0.54, *p* < 0.001)(Fig. 3), but reticulocyte was not related to percent change in haemoglobin (r = 0.26, *p* = 0.02). To determine the important variables related to increase in haemoglobin, 8 variables; 2 qualitative variables (presence of anaemia and iron supplemantaion) and 6 quantitative variables (ESA dose, erythropoietin resistance index, C-reactive protein, intact parathyroid hormone, erythrocyte creatine content and reticulocyte) were used in the multivariate logistic analysis. As a result, the erythrocyte creatine content emerged as the most independent variable associated with increase in haemoglobin (Chi-square = 6.19, *P* = 0.01) (Table 4).

**Table 3:** Comparison between patients with and without increase in haemoglobin

	^a^Increase in haemoglobin		*P* Value
	yes (*n* = 37)	no (*n* = 46)	
Age (years)	73.7 ± 14.5	70.2 ± 14.5	0.28
Gender (male/female)	22/15	34/12	0.24
Haemodialysis time (hours/week)	15.6 ± 3.6	16.2 ± 4.6	0.57
Intradialytic ultrafiltration rate (ml/hour/kg)	8.7 ± 2.7	8.9 ± 3.2	0.78
Kt/v	2.0 ± 2.0	1.7 ± 0.5	0.34
Erythrocyte creatine (μmol/gHb)	2.04 ± 0.64	1.52 ± 0.39	0.0001
**ESA dose** (units/kg/week)	125.3 ± 67.8	88.8 ± 50.2	0.006
Erythropoietin resistance index (units/kg/week/g/dL)	13.8 ± 8.7	8.4 ± 5.2	0.0008
Anaemia (present/absent)	19/18 (51%)	8/38 (17%)	0.001
Haemoglobin (g/dL)	9.73 ± 1.34	10.79 ± 0.31	0.0001
Haemoglobin at 3 months (g/dL)	10.47 ± 1.35	10.17 ± 1.03	0.25
Reticulocyte (%)	16.7 ± 4.4	14.4 ± 5.0	0.02
Haptoglobin (g/dL)	88.5 ± 51.1	97.3 ± 50.4	0.46
Transferrin saturation (%)	27.8 ± 17.1	27.8 ± 15.6	0.99
Ferritin (ng/mL)	113.6 ± 101.3	127.5 ± 111.2	0.56
Iron deficiency (present/ absent)	26/11 (70%)	21/25 (45%)	0.02
Iron supplement (present/ absent)	12/25 (32%)	10/36 (22%)	0.27
Intact parathyroid hormone (pg/mL)	160.86 ± 109.4	145.50 ± 96.27	0.49
C-reactive protein (mg/dL)	0.32 ± 0.37	0.32 ± 0.31	0.96

Data are represented as mean ± SD. *ESA* Eythropoiesis stimulating agent, *Kt/v* Urea clearance

^a^Increase in haemoglobin Haemoglobin from baseline to 3 months >0.1 g/dL

**Table 4:** Factors related to ^a^increase in haemoglobin

	Regression coefficient	Chi-square	*P* Value
**ESA dose**	**0.06**	4.96	0.03
Erythropoietin resistance index	**−0.59**	5.63	0.02
C-reactive protein	**0.49**	0.40	0.53
Intact parathyroid hormone	**0.001**	0.08	0.78
^b^**Iron supplementation**	**0.21**	0.09	0.76
^c^**Presence of anaemia**	**0.11**	0.02	0.89
Erythrocyte creatine	**−1.72**	6.19	0.01
Reticulocyte	**−0.11**	3.13	0.08

ESA Eythropoiesis stimulating agent

^a^Increase in haemoglobin Haemoglobin from baseline to 3 months >0.1 g/dL

^b^Iron supplementation Intravenous iron treatment and oral iron supplements (present/absent)

^c^Presence of anaemia Haemoglobin at baeline <10 g/dL

## Discussion

Primary mechanism of renal anaemia is a decreased erythropoietic capacity to make up for the loss of erythrocytes during chronic dialysis treatment. The most significant parameter responsible for this is an inadequate erythropoietin production by the kidney [1]. Treatment of ESA is effective in correcting anaemia and improves quality of life, exercise tolerance and left ventricular hypertrophy, which leads to better prognosis [20–25]. Therefore, it is important to establish a monitoring system to evaluate the ameliorative effectiveness of ESA in patients receiving ESA treatment. In the present study, we measured erythrocyte creatine levels with other haematologic indices in patients receiving maintenance haemodialysis on ESA treatment and found that patients with anaemia (haemoglobin <10 g/dL) had significantly higher erythrocyte creatine levels and were on higher dose of ESA administration as compared to those without anaemia (haemoglobin >10 g/dL). Moreover, erythrocyte creatine content was found to be the most important marker related to the ameliorative effectiveness of ESA at 3 months follow-up.

An excellent correlation between erythrocyte creatine content and direct measurement of red blood cell survival time by ^51^Cr method has been reported [12, 13]. In patients with normal erythropoietic capacity such as intravascular haemolysis and haemolytic anaemia, red cell production is accelerated in proportion to the amount of erythrocyte destruction, which lead to an increase in the young erythrocytes containing higher erythrocyte creatine levels [26–29]. In contrast, renal anaemia is mainly caused by decreased erythropoietic capacity due to inadequate erythropoietin production and/or hyporesponsiveness to ESA treatment [6, 30]. In haemodialysis patients with renal anaemia, the content of erythrocyte creatine is regarded as an index of erythropoiesis rather than erythrocyte age. On the basis of this concept, we measured erythrocyte creatine levels to evaluate erythropoietic capacity in patients undergoing chronic haemodialysis. Despite no significant differences in haptoglobin, transferrin saturation, ferritin, C-reactive protein and parathyroid hormone between patients with anaemia and those without anaemia, ESA dose and erythrocyte creatine levels were significantly higher in patients with anaemia compared to those without. Moreover, a significant positive relation was observed between ESA dose and erythrocyte creatine. Therefore, erythropoiesis is accelerated in response to high dose of ESA treatment in haemodialysis patients with renal anaemia due to inadequate erythropoietin production.

The optimal frequency of follow up testing in patients with anaemia is still not known. The Kidney Disease: Improving Global Outcomes (KDIGO) guidelines recommended to measure haemoglobin at least every 3 months in patients with anaemia. However, haemoglobin should be monthly checked in patients with erythropoiesis stimulating agent initiation for dose adjustments to assess its response [15]. Beguin et al. [30] reported 2 groups of respond to ESA treatment; respond within 3 months after baseline examination (early responders) and respond 3–6 months after baseline examination (late responders). In clinical practice, it is proposed that at least 1–2 months is needed to observe a significant response to ESA by monitoring the erythrocyte indices; haemoglobin or haematocrit [15, 16]. However, due to the long life-span of mature red blood cell, erythrocyte indices do not provide information of the rapid change in erythropoietic activity. Reticulocyte is the first form of red blood cell that enters circulation and reflects present erythropoiesis, but the measurement of total reticulocyte count has seldom been used in a clinical practice because erythrocyte has a rapid turn-over in the circulation (1–2 days) [9, 12]. Although the reticulocyte count was significantly higher in patients with increase in haemoglobin compared to those without in this study, reticulocyte was not related to percent change in haemoglobin. Moreover, the number of reticulocyte was not an independent variable associated with increase in haemoglobin by the multivariate logistic analysis. Therefore, there is a need to seek a reliable marker to assess the effectiveness of ESA treatment. The erythrocyte creatine content with a single blood sample measurement reflects average or cumulative erythropoiesis up to the present [18, 19]. Thus, we measured erythrocyte creatine levels and observed the change in haemoglobin for 3 months by keeping the same dose of ESA during this period. Despite no significant relation between reticulocyte and percent change in haemoglobin, erythrocyte creatine had a fair correlation with percent change in haemoglobin. Patients with a higher erythrocyte creatine content exhibited a larger improvement of anaemia 3 months later, indicating that erythrocyte creatine levels could predict accelerated erythropoiesis by ESA treatment.

Erythropoietin resistance index, a useful marker of increased mortality in patients with haemodialysis treatment, is reported to be an index of hyporesponsiveness to ESA at the time of examination. The reasons for erythropoietin hyporesponsiveness other than inadequate erythropoietin production are still not known but several factors could be involved, such as iron deficiency, inflammation, hyperparathyroidism or bone marrow dysfunction [6]. Iron deficiency, one of the main causes of hyporesponsiveness to ESA, has been widely investigated [30, 31]. In this study, incidence of iron deficiency evaluated by transferrin saturation and serum ferritin was not different between patients with favorable response (increase in haemoglobin) and those with hyporesponsiveness (no increase in haemoglobin) to ESA, because patients with absolute iron deficiency has been treated by iron supplements. Moreover, there were no differences in haptoglobin, C-reactive protein, serum ferritin and intact parathyroid hormone between patients with favorable response and those with hyporesponsiveness to ESA. Considering these factors in the multivariate analysis, erythrocyte creatine content emerged as the most important variable related to the increase in haemoglobin. Considering the report by Takemoto et al. [19], our data indicate that accelerated erythropoiesis by ESA was responsible for the improvement of anaemia and hence, erythrocyte creatine levels represent a useful marker reflecting ameliorative effectiveness of ESA.

### Clinical implications

As erythrocyte creatine content is a good indicator of the erythropoietic response to ESA, and thus early detection of therapeutic efficacy of ESA can be achieved. In patients with a low erythrocyte creatine value, dose of ESA should be increased early in the course of treatment without waiting for 3 months.

### Limitations

Three limitations of this study should be addressed. The first, this study was limited by a small number of patients. Further studies in a large group of haemodialysis patients are warranted to confirm our data. The second, the effect of ESA treatment is diminished by insufficient available iron supply [30, 31]. Absolute iron deficiency is defined as a decrease in total body iron stores and functional deficiency exhibits defective iron mobilization/utilization that cannot keep pace with the demands for the accelerated erythropoiesis regardless of the degree of iron reserve [30]. Reticulocyte haemoglobin content and high-fluorescence reticulocyte count are shown to be accurate predictors of iron-deficient erythropoiesis in patients with ESA treatment [31–33], but we did not measure reticulocyte haemoglobin content and high-fluorescence reticulocyte count to evaluate the functional iron-limited erythropoiesis. However, intravenous and oral iron supplements were given as needed to avoid possible functional iron deficiency. The Third, it has recently been proposed that the patients with haemodialysis treatment have impaired creatine homeostasis which leads to poor quality of life. Therefore, creatine supplementation is recommended in patients with haemodialysis for maintaining endogeneous creatine pools [34, 35]. Although erythrocyte creatine content is an accurate indicator of erythropoietic activity among the patient not taking creatine supplementation, further study is needed to investigate clinical significance of erythrocyte creatine in patients with end-stage renal disease on creatine supplementation.

## Conclusion

Erythrocyte creatine content, a useful indicator for erythropoietic capacity, is a reliable marker to estimate the ameliorative effectiveness of ESA in patients undergoing chronic haemodialysis.

## Data Availability

The datasets used and/or analysed during the current study are available from the corresponding author on reasonable request.

## Abbreviation

ESA: Erythropoiesis stimulating agent
